# Patient Experiences of Perioperative Care in Lumbar Spine Surgery: A Qualitative Study

**DOI:** 10.1002/nop2.70688

**Published:** 2026-07-30

**Authors:** Giorgia Petrucci, Gianluca Vadalà, Fabrizio Russo, Girolamo Maltese, Rocco Papalia, Vincenzo Denaro

**Affiliations:** ^1^ Operative Research Unit of Orthopaedic and Trauma Surgery Fondazione Policlinico Universitario Campus Bio‐Medico Rome Italy; ^2^ Università Campus Bio‐Medico di Roma Rome Italy; ^3^ Research Unit of Orthopaedic and Trauma Surgery, Department of Medicine and Surgery Università Campus Bio‐Medico di Roma Rome Italy

**Keywords:** lumbar spine surgery, orthopaedic nursing, patient experience, perioperative care, qualitative research

## Abstract

**Aim:**

To explore and describe patients' experiences throughout the perioperative pathway of lumbar spine surgery.

**Design:**

Qualitative descriptive study.

**Methods:**

A qualitative descriptive design was adopted. Participants were recruited among patients who had undergone lumbar spine surgery and were attending routine postoperative follow‐up visits approximately 1 month after surgery in an orthopaedic outpatient clinic. Semi‐structured face‐to‐face interviews were conducted in a private room. Interviews were audio‐recorded, transcribed verbatim and analysed using inductive qualitative content analysis supported by MAXQDA software. Codes were iteratively compared and grouped into subcategories and broader categories capturing key dimensions of the perioperative experience.

**Results:**

Twenty patients participated. Analysis generated 123 codes organised into 18 subcategories and four main categories: (1) the preoperative experience: pain, disability and emotional responses; (2) trust in healthcare professionals; (3) immediate postoperative relief and early recovery challenges; and (4) looking back: timing, recurrence and future concerns.

**Conclusion:**

The perioperative pathway of lumbar spine surgery represents a complex experience influenced by emotional, relational and physical factors. Trust in healthcare professionals and effective communication strongly impact perceptions of care.

**Implications for Nursing Practice:**

Understanding patients' experiences may support nurses in delivering individualised perioperative care through improved communication, patient education and emotional support across the surgical pathway. *Problem*: Limited evidence explores patients' perspectives throughout the perioperative pathway. *Findings*: Patients' experiences evolve across the surgical trajectory and are strongly influenced by communication, trust in the care team and early recovery challenges. *Impact*: Findings may inform nursing practice and perioperative care planning aimed at improving care for patients undergoing lumbar spine surgery.

**Reporting Method:**

The study adhered to COREQ guidelines.

**Patient Contribution:**

Patients contributed to the study as interview participants. They were not involved in the design, conduct, analysis or dissemination of the research.

## 1 Introduction

Low back pain is one of the leading causes of disability worldwide and represents a major public health burden (Ferreira et al. 2023). When conservative management fails, spinal surgery becomes a key therapeutic option for patients affected by degenerative, traumatic or structural spinal disorders (Evans et al. 2023). Although advances in surgical techniques and perioperative care have improved clinical outcomes, the experience of undergoing spinal surgery remains complex and demanding (Mensah et al. 2024). Beyond physical recovery, patients frequently face uncertainty, fear of complications and concerns regarding postoperative pain and functional limitations. These perioperative experiences may influence engagement in rehabilitation, satisfaction with care and overall recovery (Cooper et al. 2023).

While spinal surgery pathways are increasingly framed within patient‐centred care models, limited attention has been paid to patients' experiential perspectives throughout the perioperative process. Greater insight into these experiences is needed to inform improvements in patient‐centred perioperative care.

## 2 Background

Low back pain is a multifactorial condition frequently associated with chronic disability, reduced quality of life and significant socioeconomic burden (Alfalogy et al. 2023). For patients affected by degenerative, traumatic or structural spinal disorders that significantly compromise functional capacity, spinal surgery often represents a key therapeutic option aimed at relieving symptoms and restoring function (Evans et al. 2023).

Advances in surgical techniques, intraoperative monitoring, anaesthesia protocols and postoperative rehabilitation have progressively improved clinical outcomes. As a result, the scientific literature has primarily focused on objective clinical outcomes, including pain reduction, functional recovery and complication rates. Quantitative studies have also explored predictors of postoperative outcomes, and patient‐reported outcome measures (PROMs) have increasingly been incorporated into surgical evaluation. However, standardised outcome measures may not fully capture the complexity of patients' emotional responses, informational needs and perceptions of professional support throughout the perioperative pathway (Rodríguez‐Fuertes et al. 2023).

Beyond clinical outcomes, the experience of undergoing spinal surgery also involves significant psychological and emotional dimensions (Skarsgard et al. 2025). The preoperative phase is frequently characterised by uncertainty, fear of complications and concerns regarding postoperative pain and functional recovery (King and Hoppe 2013). Preoperative anxiety, uncertainty regarding surgical results and concerns about postoperative pain management have been associated with poorer recovery experiences (Petrucci et al. 2025).

Similarly, the early postoperative period may generate emotional vulnerability, unmet expectations and difficulties in adapting to temporary or persistent functional limitations (Sibbern et al. 2017). These aspects may directly influence patient satisfaction, adherence to postoperative recommendations and overall recovery trajectory.

Within this context, the perioperative pathway can be understood as a complex transitional experience rather than a purely clinical process. Patients are required to process large amounts of information, adjust expectations regarding recovery and navigate interactions with multiple healthcare professionals (Bougeard and Watkins 2019).

Despite the clinical relevance of these aspects, qualitative evidence exploring lived experiences of patients throughout the spinal surgery pathway remains limited. There is still limited understanding of how patients perceive the perioperative experience and how interactions with healthcare professionals influence their experiences throughout the surgical pathway. Therefore, this study aimed to explore patients' lived experiences across the spinal surgery pathway to provide insights that can be used to enhance patient‐centred perioperative care.

## 3 Study Objective

The aim of this qualitative study was to explore patients' experiences during the perioperative spinal surgery pathway within an orthopaedic context.

## 4 Methods

### 4.1 Study Design

A qualitative descriptive design was adopted. The reporting of this study was guided by the Consolidated Criteria for Reporting Qualitative Research (COREQ) checklist (Tong et al. 2007) (File S1).

### 4.2 Theoretical Framework

This study was guided by a qualitative descriptive framework as described by Sandelowski (2000). This approach explores patients' perioperative experiences while remaining close to participants' own language and descriptions of events.

### 4.3 Sampling and Recruitment

Participants were recruited using consecutive sampling among patients who underwent lumbar spinal surgery at the orthopaedic department and attended a 1‐month postoperative follow‐up visit. On the day scheduled for recruitment, the research nurse (GP) reviewed the clinical lists of the orthopaedic outpatient clinic to identify eligible patients. Eligible participants were approached face‐to‐face in the clinical setting and informed about the aim of the study. Patients who expressed interest in participating were provided with detailed information and invited to sign the written informed consent form. No eligible patients declined participation.

### 4.4 Sample Size and Power

Data collection and analysis were conducted concurrently, and recruitment continued until data saturation was achieved, defined as the point at which no new themes or relevant insights emerged from the interviews (Kerr et al. 2010). To support the assessment of saturation, a saturation grid (File S2) was used to systematically track the emergence of new codes and themes across interviews. In addition, the concept of information power (Malterud et al. 2016) was also considered throughout the recruitment and analytic process. Given the broad exploratory aim of describing the overall perioperative experience of patients undergoing lumbar spine surgery, a relatively larger sample was considered appropriate to capture a wide range of experiences and perspectives. The use of open‐ended exploratory interviews further supported the collection of sufficiently informative data relevant to the study objectives.

### 4.5 Population and Sample

The study population consisted of adult patients who underwent elective lumbar spinal surgery for degenerative conditions at the orthopaedic department and attended the 1‐month postoperative follow‐up visit. Participants included individuals who underwent different lumbar surgical procedures and presented heterogeneous demographic and clinical characteristics. Detailed participant characteristics are presented in Section 5.1.

### 4.6 Data Abstraction

Basic demographic and clinical information was abstracted during data collection, including age, sex, marital status, educational level and type of lumbar spinal surgical procedure.

### 4.7 Inclusion and Exclusion Criteria

Inclusion criteria were as follows: being 18 years or older; having undergone elective spinal surgery for degenerative conditions; having completed the immediate postoperative phase; and being able to understand and speak Italian. Patients were excluded in the presence of severe cognitive impairment or clinical conditions that could compromise their ability to participate in an interview.

### 4.8 Data Collection

Participants' availability was taken into consideration, and a one‐time face‐to‐face interview was conducted in a private room after consent. Participants were interviewed during routine surgical follow‐up visits, about 1 month after surgery in the orthopaedic outpatient clinic. Those who agreed to take part were interviewed in a private room after the clinical visit.

Semi‐structured interviews were used to generate qualitative data. One interview was conducted with each participant. All interviews were conducted by a female research nurse holding a PhD and trained in qualitative research methods. Interviews lasted between 7 and 15 min, with an average duration of approximately 10 min. No field notes were collected during the interviews. No prior therapeutic or clinical relationship existed between the interviewer and the participants. Interviews were conducted with no other persons present to facilitate open and confidential narration. An interview guide was developed based on the researcher's clinical experience and existing literature on perioperative patient‐centred care (Accardi‐Ravid et al. 2020; Davis et al. 2013; Lam et al. 2022). The guide included an opening question, exploratory questions addressing preoperative and postoperative experiences, informational needs and emotional responses, and a closing question inviting participants to add further reflections (Table 1).

**TABLE 1:** Interview guide.

1	How did you experience the preoperative phase?	
	1a	What were your main thoughts or concerns during that period?
2	Was there anything you would have liked to know more about before the surgery?	
	2a	(*If the participant asked questions before surgery*: What kind of information did you ask for?)
3	What were your feelings and emotions in the immediate postoperative period?	
	3a	Did you experience any particular difficulties or discomfort during that time?
4	How was your postoperative experience?	
	4a	Did you experience any particular difficulties or discomfort?
	4b	Was there anything you would have liked to know earlier or in more detail during the postoperative period?
5	What advice would you give to a patient who is about to undergo the same surgery?	
6	Is there anything else you would like to add that we haven't discussed?	

The interview guide was reviewed by members of the multidisciplinary research team to ensure clarity and relevance and was pilot tested with the first three participants without requiring any modifications.

### 4.9 Data Analysis

Data were analysed using a qualitative descriptive approach as described by Sandelowski (2000). Interviews were audio‐recorded and transcribed verbatim. Non‐verbal features such as pauses were not systematically transcribed, as the analysis focused primarily on the semantic content of participants' narratives, consistent with the qualitative descriptive approach. The analysis followed a four‐step process. First, transcripts were read several times to achieve immersion in the data. Second, meaningful segments of text were identified and assigned descriptive codes. Coding remained primarily descriptive and closely connected to participants' own language and accounts, consistent with the qualitative descriptive approach. Third, codes were compared across interviews and grouped into subcategories based on similarities in participants' experiences. Subcategories and categories were developed inductively through iterative comparison across transcripts. Finally, related subcategories were organised in main categories to reflect key dimensions of the patient experience across the surgical pathway. Data management and coding were supported using MAXQDA software (version 24).

Primary coding was conducted by the interviewer (GP). A second member of the research team independently reviewed the coding structure and category development. Any discrepancies were discussed within the multidisciplinary research team until consensus was reached. Representative quotations were selected to illustrate each category and were identified using anonymised participant codes (e.g., P01, P02).

### 4.10 Ethical Considerations

The study was conducted in accordance with the principles of the Declaration of Helsinki. Ethical approval was obtained from the Ethics Committee (approval number 44.24EM. CETcbm). All participants received verbal and written information about the study and provided written informed consent prior to participation. All data were anonymised and handled in accordance with applicable data protection regulations.

### 4.11 Rigour and Reflexivity

To ensure rigour, the study followed the criteria of credibility, dependability, confirmability and transferability proposed by Lincoln and Guba (Lincoln et al. 1985). Credibility was supported through repeated reading of the transcripts and systematic comparison of codes and categories across interviews. Representative quotations were used to illustrate participants' perspectives.

Dependability was ensured by clearly documenting the analytical process, including the development of codes, subcategories and categories. Data were managed using MAXQDA, which allowed transparent organisation of the coding process.

Confirmability was supported through regular discussions within the research team during data analysis to review and refine codes and categories until agreement was reached. Transferability was addressed by providing a clear description of the study context and participants.

The multidisciplinary research team included orthopaedic clinicians with extensive clinical and research experience in orthopaedic care, as well as a research nurse with experience in orthopaedic nursing, qualitative research and clinical research involving orthopaedic patients. The interviewer (GP) was a female research nurse with PhD training and previous experience in qualitative interviewing.

Reflexivity was maintained throughout the study, considering the researchers' professional backgrounds and previous clinical experience in orthopaedics and perioperative care, which could have influenced the focus on wider perioperative aspects of the surgical experience. During data collection and analysis, the research team regularly discussed potential preconceptions and their possible influence on data interpretation. To minimise the influence of individual preunderstandings, the interview guide was designed using open‐ended, exploratory questions that allowed participants to freely describe their experiences and concerns related to lumbar spine surgery. Furthermore, coding decisions and category development were continuously discussed within the multidisciplinary research team to maintain close adherence to participants' narratives and enhance reflective awareness throughout the analytical process. Participant validation (member checking) was not undertaken as part of the study design.

## 5 Findings

### 5.1 Participants

Twenty participants undergoing lumbar spine surgery were included in this study, comprising 9 females (45%) and 11 males (55%). The mean age of patients was 54 years. The most frequently performed procedure was discectomy, which was carried out in 13 cases (65%), while transforaminal lumbar interbody fusion (TLIF) was performed in seven cases (35%). The L4–L5 level was the most treated spinal segment, being involved in 13 procedures (65%). Other treated levels included L3–L4 in five cases (25%), L5–S1 in four cases (20%) and L2–L3 in one case (5%). Most surgical procedures involved a single spinal level, whereas three cases (15%) required surgery at two spinal levels. In addition, one patient (5%) underwent revision surgery due to recurrent disc herniation at the L4–L5 level. Detailed participant characteristics are presented in Table 2.

**TABLE 2:** Participant characteristics.

Characteristic	*n* (%)
Sex	
Female	9 (45)
Male	11 (55)
Age group (years)	
30–49	6 (30)
50–69	13 (65)
≥ 70	1 (5)
Marital status	
Married	15 (75)
Single	3 (15)
Divorced	2 (10)
Educational level	
ISCED 2	5 (25)
ISCED 3	10 (50)
ISCED 6	5 (25)
Surgical procedure	
Discectomy	13 (65)
TLIF	7 (35)
Surgical levels involved	
L2–L3	1 (5)
L3–L4	5 (25)
L4–L5	13 (65)
L5–S1	4 (20)
Two‐level surgery	3 (15)
Revision surgery	1 (5)

Abbreviations: ISCED, international standard classification of education; TLIF, transforaminal lumbar interbody fusion.

### 5.2 Description of Categories

The analysis generated 123 initial codes, which were iteratively compared and grouped according to similarities and recurring patterns. All the codes were organised into 18 descriptive subcategories, which were subsequently clustered into four main categories representing key dimensions of the patient experience. The codes' system is presented in Table 3.

**TABLE 3:** Main themes, subcategories and representative codes emerging from the qualitative analysis of patients' perioperative experiences following lumbar spine surgery.

System of codes (*n* = 123)	*N*
The preoperative experience: pain, disability and emotional responses	*n* = 51
Adequate preoperative information	14
Severe preoperative physical pain	14
Fear of permanent disability	11
Preoperative emotional calmness	4
Fear related to surgery	3
Relief after surgery scheduling	2
Impact of condition on work and lifestyle	2
Perceived psychological component of pain	1
Trust in healthcare professionals	*n* = 24
Highly positive care experience	10
Trust in surgical team	5
Reassurance through professional presence	5
Clear postoperative instructions	4
Immediate postoperative relief and early recovery challenges	*n* = 34
Distressing awakening experience	3
Unexpected slow recovery	4
Postoperative functional difficulties	10
Postoperative relief	17
Looking back: timing, recurrence and future concerns	*n* = 14
Advice not to delay surgery	10
Concern about possible recurrence	2
Uncertainty about surgical details	2

**FIGURE 1:**
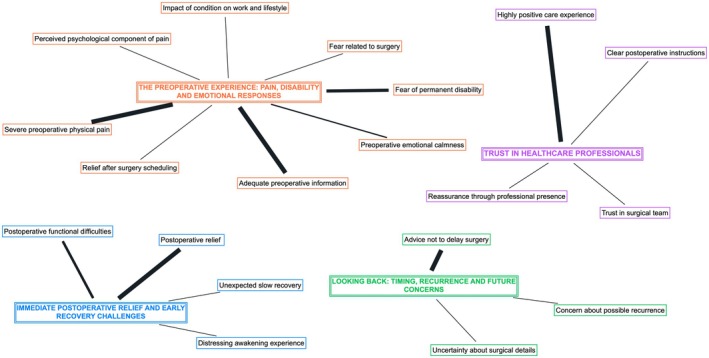
Conceptual map of patients' experiences across spinal surgery generated using MAXQDA. The map illustrates the relationships between the four main categories and their associated subcategories. Connecting lines indicate conceptual links between themes, while line thickness reflects the relative strength of the relationships identified in the data.

The four main categories were conceptually organised following a chronological‐evolutionary structure, reflecting the trajectory of the patients' experiences across the surgical pathway, from the preoperative phase to postoperative recovery and subsequent reflections on the surgical pathway. The resulting categories were: (1) the preoperative experience, (2) trust in healthcare professionals, (3) immediate postoperative relief and early recovery challenges and (4) reflections and future‐oriented concerns. The relationships between the identified categories and subcategories are illustrated in Figure 1, which presents a conceptual map generated using MAXQDA. The map visually represents the organisation of the data, showing how the main categories are connected to their respective subcategories, which emerged from the coding process.

Each main category is represented by a central node, from which the related subcategories branch out, illustrating the different dimensions of the patients' experiences identified in the analysis. The connections between nodes are represented by connecting lines that indicate the conceptual relationships between categories and subcategories.

The thickness of the connecting lines reflects the strength of the relationship identified during the analysis. Thicker lines indicate stronger or more prominent relationships within the dataset, reflecting themes that emerged more consistently or were more strongly connected within participants' narratives. Conversely, thinner lines represent weaker or less frequent associations between categories and subcategories.

#### 5.2.1 The Preoperative Experience: Pain, Disability and Emotional Responses

The preoperative phase was characterised by intense physical suffering and significant functional limitations. Many participants described experiencing severe preoperative pain, which strongly affected their mobility and daily life, often interfering with work activities and normal routines. Some individuals reported being unable to sleep or perform basic movements due to the intensity of the pain.

I experienced severe pain that could not be controlled with medication. It caused considerable difficulties both at work and in my daily life. (P 20)

Alongside the physical burden, patients frequently expressed fear of permanent disability, particularly concerns about losing the ability to walk or fully recover neurological function.

My main concern was that I could no longer walk, and I was afraid of losing the ability to walk. (P 7)

Despite these concerns, several participants reported receiving adequate preoperative information, which helped them better understand the procedure and increased their sense of reassurance. A few individuals also described a state of relative emotional calmness prior to surgery, often linked to trust in the medical team.

I was always well informed. During each visit, the healthcare professionals explained the causes of my condition, why I was experiencing pain, and the possible consequences. Every question I had was always answered. (P 18)

For some patients, the moment when surgery was finally scheduled represented a turning point, producing a sense of relief after surgery scheduling following a prolonged period of uncertainty and suffering.

The waiting period was quite long, and during that time I suffered a lot, both physically and psychologically. However, when I was informed about the date of the surgery and the planned hospital admission, my psychological condition improved significantly. By the time I was admitted to the hospital, I felt very calm and was even looking forward to it. (P 1)

#### 5.2.2 Trust in Healthcare Professionals

Participants frequently reported a strong sense of trust in healthcare professionals. Many described a highly positive care experience, emphasising the professionalism, availability, and supportive attitude of the healthcare staff. The presence of competent and attentive professionals contributed to feelings of safety and reassurance during both the preoperative and postoperative phases.

Everything was managed with great attention and professionalism. I received consistent care and regular follow‐up visits, and I was very impressed by the quality of the service. The environment was comfortable and the healthcare staff were highly professional. (P11)

Trust in the surgical team was also frequently mentioned. Participants expressed confidence in the surgeons and healthcare staff, which helped reduce anxiety and strengthened their willingness to undergo the procedure.

I had never undergone general anaesthesia before, so I was initially afraid of the anaesthesia. However, I trusted the doctors completely because they had made such a positive impression on me, and I eventually had no real fear about the surgery itself (…) Despite my initial fear of hospitals and the emotional impact of undergoing my first surgery, the experience turned out to be extremely positive. I felt comfortable throughout the process and even looked forward to returning to greet the healthcare professionals. (P1)

Several patients highlighted how the clear explanations provided by healthcare professionals fostered a sense of confidence and understanding regarding their condition and treatment.

Additionally, the professional presence of the healthcare team played an important role in reassuring patients during hospitalisation.

Whenever I had a difficulty, the healthcare professionals were always there for me. I never felt abandoned and was consistently supported. (P14)

Participants reported feeling supported and guided throughout the process. Clear postoperative instructions were also appreciated, as they helped patients feel more prepared to manage the recovery period after discharge.

The healthcare professionals provided me with a lot of information and explained clearly how I should move after the surgery, including how to use the brace and which movements I needed to be careful about. (P2)

#### 5.2.3 Immediate Postoperative Relief and Early Recovery Challenges

Participants described varied experiences during the immediate postoperative period. While several individuals reported a sense of relief after surgery, others described initial discomfort and challenges during the early stages of recovery.

After the surgery, I felt reborn. Once I was able to get out of bed, wear the brace, and start walking again, I felt like a new person. Of course, there were still some limitations, but the pain was gone and everything felt completely different. (P5)

Some participants reported a distressing awakening experience following anaesthesia, characterised by confusion, discomfort or emotional distress immediately after the procedure. In a few cases, patients also described an unexpectedly slow recovery during the first days after surgery, which generated concerns about their postoperative progress.

Functional limitations were frequently mentioned during the early recovery phase. Many participants reported difficulties in performing daily activities, particularly related to mobility and movement restrictions after surgery.

During the first few days after surgery, I experienced several difficulties with basic daily activities, such as going to the bathroom, washing myself, and even getting out of bed. (P7)

Despite these initial difficulties, most of participants described a significant sense of relief after surgery. Many reported a reduction in preoperative pain and expressed a feeling of improvement shortly after the procedure, which contributed to a more positive perception of the overall recovery process.

I started to feel more confident because the worst had passed and I could begin to see signs of improvement. (P3)

#### 5.2.4 Looking Back: Timing, Recurrence and Future Concerns

When reflecting on their overall experience, some participants expressed lingering concerns about the future. Some participants reported uncertainty regarding the technical aspects of the surgical procedure, stating that certain details of the surgery had not been fully clear to them despite the information provided.

I knew that the surgery would involve screws, but I didn't know how many they would actually use. (P7)

Another concern reported by several participants was the possibility of recurrence of their condition. Although the surgery was generally perceived as effective, some individuals still worried about the potential return of symptoms over time.

It's true that the surgery went well, but no one told me that there couldn't be a recurrence. (P13)

Despite these concerns, many participants reflected positively on their experience and emphasised the importance of not delaying surgery. Based on their personal experience, several suggested that undergoing the procedure earlier could have prevented prolonged suffering and functional limitations.

The surgery should be done as soon as possible, without waiting. I postponed it for too long because of fear, and that was a mistake. The surgery changed my life. (P16)

## 6 Discussion

This study provides insights into how patients experience the perioperative pathway following lumbar spine surgery, highlighting that it is influenced not only by physical symptoms but also by emotional responses, educational needs and relationships with healthcare providers. These findings underline the multidimensional nature of the perioperative experience and highlight the importance of adopting a more patient‐centred approach to spinal surgical care. Overall, patients described a complex experience characterised by severe preoperative distress, expectations regarding surgery and a postoperative phase that brought both relief and new challenges during recovery. The preoperative phase was strongly characterised by severe pain and functional limitations that significantly impacted participants' daily activities and quality of life. Many participants described long periods of persistent pain that interfered with work, mobility and social life, ultimately leading them to consider surgery as a necessary step after the failure of conservative treatments. Similar experiences have been reported in previous qualitative studies of patients undergoing spinal surgery, in which subjects described surgery as a last resort following periods of severe pain and functional disability (van der Horst et al. 2019). In addition to physical pain, emotional responses played an important role in shaping the preoperative experience. Participants frequently reported fears related to the potential consequences of surgery, including concerns about mobility and long‐term disability. More specifically, several participants described fears related to neurological impairment, paralysis, loss of independence and the possibility of permanent functional decline, aspects that may be particularly relevant in lumbar spine surgery. Furthermore, the chronic and disabling nature of lumbar spinal conditions appeared to contribute to a strong psychological burden, often characterised by anxiety, emotional distress and uncertainty regarding future functional recovery (Choi et al. 2021). These elements may contribute to making the perioperative experience of lumbar spine surgery particularly complex and emotionally demanding. Our findings extend previous evidence by showing the close connection of emotional concerns and perceptions of future autonomy and quality of life. Information and therefore effective communication emerged as a key element influencing patient experience throughout the surgical process. Most participants reported satisfaction with the communication methods, also linking it to a feeling of calm. To a lesser extent, some described residual uncertainty regarding technical and specific aspects of the surgical procedure. Conversely, another study has shown that communication and the provision of information to patients were lacking (McCarthy et al. 2020). Communication with healthcare providers therefore plays a crucial role in the patient experience. Our participants frequently emphasised the importance of clear explanations, professional expertise and a supportive attitude from the team. These elements helped build trust and helped patients feel reassured throughout the perioperative process. These findings reinforce the importance of patient‐centred communication strategies in reducing uncertainty and promoting emotional reassurance throughout the perioperative pathway. However, despite the physical and emotional challenges of lumbar spine surgery, many participants reported a high level of satisfaction with the perioperative care they received. Feelings of trust, perceived professional competence, availability of healthcare professionals and supportive communication appeared to positively influence patients' overall perceptions of care. The results suggest that the relational and communicative aspects of perioperative care, particularly supportive relationships with healthcare professionals, may positively influence the patient's surgical experience beyond clinical outcomes, despite physical distress and recovery difficulties. Participants frequently described trust in the surgical team, reassurance and confidence in professional expertise as central components of a positive perioperative experience. In this regard, our findings resonate with recent reflections on patient‐centred care, suggesting that responding to patients' needs may also involve providing guidance, taking professional responsibility and fostering trust in clinical expertise throughout the care pathway (Pilnick 2023). Previous research exploring the experiences of patients undergoing spinal surgery has similarly identified communication and supportive interactions with the team as central components of a positive care experience (Davis et al. 2013).

The postoperative phase has often been described as a turning point in the patient experience. Many participants reported a substantial reduction in the intense pain they experienced before surgery, often describing the procedure as life changing. However, recovery was not always as smooth as expected. Some participants described temporary functional limitations, emotional distress or a slower‐than‐expected recovery process. This is consistent with another study in which patients described the postoperative period as a complex phase requiring adaptation and coping strategies (Accardi‐Ravid et al. 2020). Regarding postoperative experiences, several participants expressed concerns about the recovery process and the possibility of recurrence or long‐term complications. These concerns appear to be common among spinal surgery patients and are often related to uncertainties regarding the postoperative recovery process. This is a particularly meaningful aspect among our participants, reflecting concerns about returning to a condition previously associated with severe pain, disability and compromised quality of life. Finally, many participants emphasised the importance of not delaying surgery once symptoms had significantly impacted their quality of life. In their accounts, postponing surgery was often associated with prolonged suffering and functional limitations. At the same time, some participants expressed ongoing concerns about the possibility of recurrence or future complications. These reflections highlight how patients continue to interpret and reevaluate their surgical experience even after the procedure. Such retrospective perspectives provide valuable insights into how patients interpret their experience and can help guide decision‐making processes for people undergoing similar surgical pathways. These findings also suggest that fear and uncertainty prior to surgery may contribute to delaying treatment, potentially prolonging physical suffering and functional impairment. Greater attention to emotional support and preoperative counselling may help patients navigate surgical decision‐making more confidently and at an earlier stage of the disease trajectory.

In conclusion, the results of this study contribute to an understanding of the emotional, informational and relational dimensions of the pathway to spinal surgery and support the need for more holistic and patient‐centred perioperative care models.

### 6.1 Strength and Limitations

The qualitative descriptive design allowed for the exploration of patient experiences throughout the perioperative lumbar spine surgery pathway, capturing perspectives not fully captured in quantitative outcome measures. The use of face‐to‐face semi‐structured interviews allowed participants to describe their experiences in their own words, generating descriptive accounts directly relevant to clinical practice. Data collection and analysis were conducted simultaneously, allowing for the exploration of emerging themes during the recruitment process and supporting the achievement of data saturation. The use of qualitative data analysis software (MAXQDA) facilitated the systematic organisation of the coding process and improved data transparency and sharing. Multi‐team review of the categories strengthened analytical credibility and supported reflexive discussion during data interpretation.

Despite these strengths, some limitations must be acknowledged. The study was conducted at a single orthopaedic centre, which may limit the applicability of the findings to other healthcare settings or patient populations.

Another limitation concerns the timing of the interviews, which were conducted after surgery. Participants' reflections on the preoperative phase may therefore have been influenced by their postoperative experiences and the retrospective reinterpretation of their surgical pathway. Furthermore, the relatively short duration of the interviews (approximately 7–15 min) may have limited the depth and richness of participants' accounts. However, the interviews generated sufficient information to address the study aim and achieve information power.

### 6.2 Recommendations for Further Research

Future research using longitudinal qualitative models could provide deeper insights into how patient perceptions evolve across the different phases of the surgical pathway, from preoperative decision‐making to long‐term postoperative recovery. Following patients over time would allow for a more comprehensive understanding of how expectations, emotional responses and information needs change throughout the perioperative pathway.

Furthermore, future studies could adopt mixed approaches that combine qualitative exploration with quantitative clinical outcome measures. Integrating patient narratives with clinical indicators and PROMs could help identify how experiential dimensions, such as trust in healthcare providers, expectations or perceived support, are associated with recovery, pain reduction and functional outcomes.

Finally, the application of research conducted in different healthcare settings and the inclusion of larger and more diverse patient populations could also help strengthen the transferability of findings and support the development of patient‐centred perioperative care strategies in spine surgery.

### 6.3 Implications for Policy and Practice

The findings underscore the importance of providing clear, consistent and accessible information throughout the surgical pathway. Nurses and healthcare professionals play a key role in ensuring patients receive understandable explanations about the surgical procedure, the expected recovery process and postoperative self‐management. Strengthening preoperative education can help reduce uncertainty and support patients' emotional preparation for surgery.

Furthermore, the findings emphasise the central role of trust and supportive relationships between patients and healthcare professionals. Participants often associated positive care experiences with the professionalism, availability and communication skills of the healthcare team. This suggests that promoting patient‐centred communication and maintaining a supportive presence during hospitalisation can help improve patients' sense of safety and trust during the perioperative period.

Structured perioperative education programmes and nurse‐led interventions may be useful strategies to address these needs and improve patient‐centred care in spine surgery.

## 7 Conclusion

This study highlights that the perioperative experience of lumbar spine surgery extends beyond clinical recovery and is strongly influenced by emotional responses, information needs and relationships with healthcare providers. Patients described a pathway characterised by severe preoperative distress, postoperative relief and ongoing reflections on recovery and potential recurrence. Trust in the health care team and open communication were key elements influencing patients' perceptions of care. The findings also highlight the importance of timely support and decision‐making assistance throughout the surgical pathway. Understanding these dimensions can support healthcare professionals in developing more patient‐centred perioperative pathways.

## Author Contributions

Giorgia Petrucci: conceptualisation, data curation, formal analysis, investigation, methodology, software, validation, visualisation, writing – original draft. Gianluca Vadalà: conceptualisation, resources, supervision, project administration, funding acquisition, writing – review and editing. Fabrizio Russo: resources, supervision, funding acquisition, project administration, software, validation, writing – original draft. Girolamo Maltese: data curation, writing – original draft. Rocco Papalia: project administration, resources, funding acquisition, writing – review and editing. Vincenzo Denaro: project administration, resources, funding acquisition, writing – review and editing.

## Funding

This study was funded by the European Union—Next Generation EU—NRRP M6C2‐Investment 2.1 Enhancement and Strengthening of Biomedical Research in the NHS (PNRR‐MAD‐2022‐12376692, CUP: F83C22002470001; PNRR‐MCNT2‐2023‐12378359, CUP: F83C24000500006).

## Disclosure

AI/writing assistance statement: No artificial intelligence (AI) or AI‐assisted technologies were used in the writing, analysis or preparation of this manuscript.

The authors agree to take responsibility for ensuring that the choice of statistical approach is appropriate and is conducted and interpreted correctly as a condition to submit to the Journal.

## Ethics Statement

Ethical approval was obtained from the Ethics Committee of the Fondazione Policlinico Universitario Campus Bio‐Medico. The study received formal approval following the Ethics Committee session held on 19 April 2023 (approval number 44.24EM. CETcbm).

## Conflicts of Interest

The authors declare no conflicts of interest.

## Supporting information


**File S1:** COREQ (COnsolidated criteria for REporting Qualitative research) checklist.

**File S2:** Saturation grid.

## Data Availability

The data supporting the findings of this study are available from the corresponding author upon reasonable request.
