# Supplementary material for: Patient Experiences of Perioperative Care in Lumbar Spine Surgery: A Qualitative Study
Source: Nurs Open. 2026 Jul 30;13(8):e70688. doi: 10.1002/nop2.70688 (PMC13420206; doi:10.1002/nop2.70688)
Supplement: Supplementary file 2 — File S2: Saturation grid. [file NOP2-13-e70688-s002.docx]

Supplementary file 2: Saturation grid

| **Patient** | **P 1** | **P 2** | **P 3** | **P 4** | **P 5** | **P 6** | **P 7** | **P 8** | **P 9** | **P 10** | **P 11** | **P 12** | **P 13** | **P 14** | **P 15** | **P 16** | **P 17** | **P 18** | **P 19** | **P 20** |
| --- | --- | --- | --- | --- | --- | --- | --- | --- | --- | --- | --- | --- | --- | --- | --- | --- | --- | --- | --- | --- |
| THE PREOPERATIVE EXPERIENCE: PAIN, DISABILITY AND EMOTIONAL RESPONSES |  |  |  |  |  |  |  |  |  |  |  |  |  |  |  |  |  |  |  |  |
| Adequate preoperative information | 1 | 2 | 2 |  | 1 | 1 |  | 1 | 1 |  |  |  |  | 1 |  |  | 1 | 2 |  | 1 |
| Severe preoperative physical pain | 1 |  | 1 | 1 | 1 | 1 | 1 | 1 |  |  | 1 |  |  | 2 | 1 |  |  | 1 | 1 | 1 |
| Fear of permanent disability |  | 1 | 1 |  | 2 |  | 1 |  |  | 2 |  | 1 | 1 |  |  |  |  |  | 1 | 1 |
| Preoperative emotional calmness |  |  |  | 1 |  |  |  |  | 2 |  |  | 1 |  |  |  |  |  |  |  |  |
| Fear related to surgery |  |  | 1 |  |  |  |  |  |  |  |  |  |  |  |  | 1 | 1 |  |  |  |
| Relief after surgery scheduling | 1 |  |  |  |  |  |  |  |  |  |  |  |  |  |  |  |  | 1 |  |  |
| Impact of condition on work and lifestyle |  |  |  |  |  | 1 |  |  |  |  |  |  |  |  |  |  |  |  |  | 1 |
| Perceived psychological component of pain | 1 |  |  |  |  |  |  |  |  |  |  |  |  |  |  |  |  |  |  |  |
| TRUST IN HEALTHCARE PROFESSIONALS |  |  |  |  |  |  |  |  |  |  |  |  |  |  |  |  |  |  |  |  |
| Highly positive care experience | 1 |  |  |  | 2 | 2 | 1 |  | 1 |  | 1 |  |  |  | 1 |  | 1 |  |  |  |
| Trust in surgical team | 1 | 1 | 1 | 2 |  |  |  |  |  |  |  |  |  |  |  |  |  |  |  |  |
| Reassurance through professional presence | 1 |  |  |  |  |  |  |  |  |  | 1 | 1 |  | 1 |  |  |  | 1 |  |  |
| Clear postoperative instructions | 1 | 1 |  |  |  |  |  |  |  |  |  | 1 |  |  |  |  |  | 1 |  |  |
| IMMEDIATE POSTOPERATIVE RELIEF AND EARLY RECOVERY CHALLENGES |  |  |  |  |  |  |  |  |  |  |  |  |  |  |  |  |  |  |  |  |
| Distressing awakening experience | 1 | 1 |  |  |  |  |  |  |  |  |  |  |  |  |  |  |  |  | 1 |  |
| Unexpected slow recovery | 1 |  |  |  | 1 |  |  |  |  | 1 |  |  |  |  |  |  |  |  |  | 1 |
| Postoperative functional difficulties | 2 |  |  |  |  |  | 1 | 1 |  | 1 |  | 1 |  | 2 | 1 |  |  | 1 |  |  |
| Postoperative relief | 2 |  | 1 | 1 | 1 | 1 | 1 | 1 | 1 |  | 1 | 1 | 1 | 1 |  | 2 |  | 1 |  | 1 |
| LOOKING BACK: TIMING, RECURRENCE AND FUTURE CONCERNS |  |  |  |  |  |  |  |  |  |  |  |  |  |  |  |  |  |  |  |  |
| Advice not to delay surgery |  |  |  | 1 |  |  |  | 1 |  | 1 |  | 2 |  | 1 |  | 1 |  | 1 | 1 | 1 |
| Concern about possible recurrence |  |  |  |  |  |  |  |  |  |  |  |  | 1 |  |  |  |  |  | 1 |  |
| Uncertainty about surgical details |  |  |  |  |  |  | 1 |  | 1 |  |  |  |  |  |  |  |  |  |  |  |
